# Acoustic Diversity in *Zhangixalus lishuiensis*: Intra-Individual Variation, Acoustic Divergence, and Genus-Level Comparisons

**DOI:** 10.3390/ani15233493

**Published:** 2025-12-04

**Authors:** Jia-Jun Hao, Zhi-Qiang Chen, Hua-Li Hu, Jian-Guo Cui, Guo-Hua Ding

**Affiliations:** 1 Laboratory of Amphibian Diversity Investigation, College of Agriculture and Biotechnology, Lishui University, Lishui 323000, China; haojune@foxmail.com; 2 Yueqing Agricultural and Rural Bureau, Yueqing 325600, China; zqchen960717@foxmail.com; 3 Administration Center of Zhejiang Jiulongshan National Nature Reserve, Suichang, Lishui 323300, China; hualihu@njfu.edu.cn; 4 Chengdu Institute of Biology, Chinese Academy of Sciences, Chengdu 610041, China; cuijg@cib.ac.cn

**Keywords:** bioacoustics, call structure, call variation, species identification, tree frog

## Abstract

Advertisement calls play a crucial role in anuran species recognition and mate selection, yet acoustic characteristics of *Zhangixalus lishuiensis* remain undescribed. This study presents the first detailed acoustic analysis of advertisement calls in *Zhangixalus lishuiensis* from Lishui, Zhejiang Province, China. Based on recordings from 13 males collected in the field during the breeding season, we documented a hierarchical call structure consisting of three note types (Initial Note, Middle Note, and Short Note) organized into two note groups (Note Group 1 (NG1) and Note Group 2). Four structural variants of NG1 were identified, revealing intra-individual acoustic diversity within the population. Compared to the closely related *Z. zhoukaiyae* from the Dabie Mountains population, male *Z. lishuiensis* produced calls with significantly longer temporal parameters but lower dominant frequency, despite overall structural similarity, indicating acoustic divergence between these taxa. Comparative analysis across eleven *Zhangixalus* species revealed substantial variation in call durationand dominant frequency. These findings enrich the bioacoustic characterization of *Zhangixalus* and establish a foundation for acoustic-based species identification, monitoring, and taxonomic studies in this genus.

## Simple Summary

Frogs use advertisement calls during breeding season to attract mates and establish territories. Each species has a unique call pattern, like a vocal signature. In this study, we recorded the calls of male *Zhangixalus lishuiensis* and discovered that the frog organizes its calls into complex structures with three distinct note types, and can produce five different call variants. Compared to the closely related *Z. zhoukaiyae*, *Z. lishuiensis* produced lower-frequency calls with longer durations and intervals. By comparing eleven *Zhangixalus* species, we found considerable variation in call characteristics across the genus. Our findings provide essential baseline acoustic data for identifying, monitoring, and conserving these tree frogs.

## 1. Introduction

In anuran amphibians, bioacoustic information plays a critical role in biodiversity surveys [1,2,3] and behavioral studies [4,5,6]. Among anuran vocalizations, male advertisement calls, which are produced during the breeding season, are particularly important, serving essential functions in mate attraction [4], species recognition [7,8], and male-male communication [9,10]. These calls exhibit considerable acoustic complexity and plasticity in response to various environmental factors [11,12]. Owing to their species specificity and functional diversity, advertisement calls not only provide valuable insights into reproductive ecology and behavioral adaptations but also serve as reliable diagnostic characters for distinguishing taxa, including cryptic species that are otherwise difficult to differentiate morphologically. As demonstrated in comprehensive taxonomic revisions that integrate molecular, morphological, and acoustic data [13], bioacoustic datasets have become essential complements to other lines of evidence in anuran biodiversity surveys and systematic research [1,14].

The taxonomic utility of bioacoustic data depends on rigorous analysis and standardized description of acoustic parameters. The analysis of temporal and spectral parameters of advertisement calls has become increasingly important in anuran taxonomy, being incorporated as diagnostic features in species descriptions [14,15]. For a standardized description of anuran advertisement calls, “call”, “note”, and “pulse” are established as fundamental units. According to Köhler et al. [16], “notes” function as subunits of “calls” and can be arranged in patterns to form different note types. These note types compose simple or complex calls, with complex calls consisting of note groups whose repetition constitutes note series. This standardized terminology helps minimize inconsistencies in characterizing advertisement calls across different studies.

Tree frogs are generally characterized as frogs who spend most of their lifespan in arboreal habitats like trees. Within the order Anura and the family Rhacophoridae, the genus *Zhangixalus* was split from *Rhacophorus* in 2019 [17]. The genus is primarily distributed in East Asia and Southeast Asia; and as of November 2025, 46 species are currently recognized within the genus *Zhangixalus* [18]. Of the 46 species, 32 of them are currently found in China and 20 of those are endemic to China [19]. Prior to recent studies, detailed acoustic analyses of advertisement calls have been published for only six *Zhangixalus* species: *Z. chenfui* [20], *Z. dennysi* [21], *Z. dugritei* [20], *Z. omeimontis* [22], *Z. pinglongensis* [23], and *Z. zhoukaiyae* [24], all from China. Recent comparative studies have expanded this dataset to include four Southeast Asian species (*Z. achantharrhena*, *Z. dulitensis*, *Z. faritsalhadii*, and *Z. prominanus* [25]), bringing the total to ten species with detailed acoustic characterizations. In addition, call recordings for 12 *Zhangixalus* species have been deposited in online bioacoustic databases [26,27,28], though most of these lack the comprehensive analyses of temporal and spectral parameters necessary for taxonomic comparisons.

*Z. lishuiensis* (Liu, Wang, and Jiang, 2017) and *Z. zhoukaiyae* (Pan, Zhang, and Zhang, 2017) are two closely related *Zhangixalus* species distributed in eastern China. *Z. zhoukaiyae* was described in March 2017 [29], while *Z. lishuiensis* was published online in May 2017 [30]. Previous phylogenetic studies have revealed exceptionally low genetic divergence between the two taxa. Based on mitochondrial 16S rRNA gene sequences, genetic distances of approximately 1.1% [31] and 1.6% [32] have been reported, with Liu et al. [33] estimating only 0.9% divergence using a combined dataset of three mitochondrial genes (12S rRNA, tRNA-Val, and 16S rRNA). Moreover, Brakels et al. [32] noted the lack of reliable diagnostic morphological characters to differentiate them. These findings led some researchers to suggest that *Z. lishuiensis* and *Z. zhoukaiyae* may represent a single species [31]. However, recent phylogenetic reconstruction combined with morphological comparisons by Pan et al. [34] continued to treat the two taxa as distinct species.

Given the lack of taxonomic consensus, the relationship between *Z. lishuiensis* and *Z. zhoukaiyae* remains unresolved. In the absence of conclusive evidence, the two taxa are maintained as separate species following their original descriptions. To resolve this uncertainty, additional evidence is needed to definitively establish their taxonomic status. Bioacoustic data have proven particularly valuable in resolving taxonomic uncertainties within morphologically cryptic anuran species complexes, potentially providing decisive diagnostic characters even when genetic divergence in mitochondrial DNA is relatively low (e.g., <3% in *Limnonectes*) [35]. While advertisement calls of *Z. zhoukaiyae* have been documented [24], the acoustic characteristics of *Z. lishuiensis* remain unknown. Therefore, this study aims to (1) describe in detail the structural organization of advertisement calls in *Z. lishuiensis*, (2) compare its acoustic features with those of *Z. zhoukaiyae* and assess the potential taxonomic implications, and (3) synthesize acoustic data across *Zhangixalus* species to understand call diversity within this genus.

## 2. Materials and Methods

### 2.1. Study Site and Recordings

**Figure 1:**
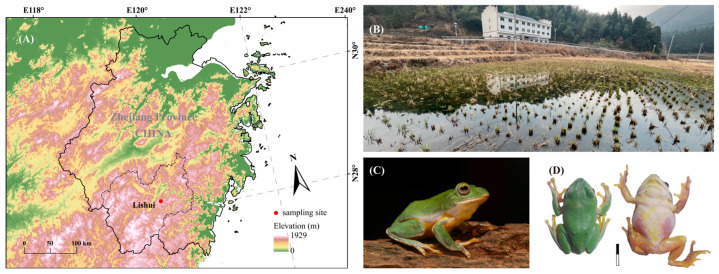
Sampling site and morphology of *Zhangixalus lishuiensis*. (**A**) Geographic location of the sampling site in Lishui, Zhejiang Province, China. (**B**) Habitat photograph of the sampling site (Photo by Yu-Fan WANG). (**C**) Lateral view of *Z. lishuiensis* (call voucher: ZL200406_001, snout-vent length = 36.2 mm). (**D**) Dorsal (**left**) and ventral (**right**) views of the same specimen. Scale bar = 1 cm. Photos in (**C**,**D**) by Guo-Hua DING.

*Z. ishuiensis* is a small tree frog that breeds in early spring, with males constructing foam nests in muddy substrates. Nests are typically built as oval chambers 5–7 cm deep, excavated in soft soil or beneath grass roots, where males vocalize [30]. In early April 2020, the 13 nests of male *Z. lishuiensis* were located in muddy soil along field ridges or under grass cover in the Liandu Fengyuan Provincial Nature Reserve, Lishui, Zhejiang, China (28.1938° N, 119.8269° E; elevation: 1038 m) (Figure 1A). Nests were spaced approximately 5 m apart, with typically one male per nest. Audio recordings were made near the field ridges of the rice paddy habitat at this location (Figure 1B). Recordings were made using a Sony PCM-A10 digital recorder (LPCM, 44.1 kHz/16 bit, “.wav” format, Minato City, Japan) with an internal microphone mounted on a tripod and placed within 50 cm of each nest. Each recording session lasted 5–10 min, and recordings were collected over three nights. Environmental conditions during recordings averaged 7.4 ± 0.3 °C air temperature and 43.5 ± 10.2 dB ambient noise.

Due to sampling constraints and the need to minimize destructive disturbance to the foam nests of *Z. lishuiensis*, only three males were collected after acoustic recordings. These individuals corresponded directly to the males whose calls had been recorded. Their snout–vent length (SVL) was measured using a digital caliper (CD-6, ±0.02 mm, Mitutoyo, Kawasaki, Japan), ranging from 34.2 to 36.2 mm. Their body mass was measured using a portable electronic digital scale (DS-16, ±0.01 g, Shenzhen Saint Diamond Technology Co., Ltd., Shenzhen, China), ranging from 3.25 to 3.97 g. Photographs of a captured *Z. lishuiensis* individual were taken, including lateral (Figure 1C), dorsal, and ventral views (Figure 1D). All individuals were released at their capture sites after measurements and photographic documentation.

### 2.2. Acoustic Analysis

Thirteen audio files, each representing one male individual, were preprocessed in Cool Edit Pro 2.1, converting stereo to mono and applying noise reduction (40 dB, FFT size = 1024 points), and subsequently analyzed using Praat 6.4.27 [36]. Spectrograms were generated with a 0–4000 Hz frequency range, 10 ms window length, and 35 dB dynamic range. Following Köhler et al. [16], the units and subunits of advertisement calls were classified and numbered. Preliminary analysis revealed that advertisement calls of *Z. lishuiensis* are hierarchically organized, with notes as the basic subunits, note groups as the primary structural units, and note series as higher-order structures composed of note groups. Each individual contributed 5–43 calls to the analysis. Acoustic parameters were extracted from oscillograms and spectrograms generated for each call. Temporal parameters included note duration (ND), duration of note group, duration of note series, call duration (CD), intervals between note groups, and call interval (CI). Additionally, the number of note groups within each note series was recorded. The spectral parameter measured was the dominant frequency (DF) at both note and call levels.

Initial visual inspection of oscillograms identified four potential note types (Note 1–4) within each call. Note 1 and Note 2 were singular, non-repetitive notes, while Note 3 and Note 4 were repetitive. From 13 audio recordings, we selected 8 files containing complete calls that included all four note types. From each of these 8 files, 5 complete calls were randomly selected as replicates for comparative analysis of note types. For the repetitive note types (Note 3 and Note 4), individual notes within each call were consecutively numbered (e.g., 3-1, 3-2, 3-3, 3-4, 3-5 for Note 3; 4-1, 4-2, 4-3, 4-4, 4-5 for Note 4). To standardize comparisons during this preliminary classification stage, only the first 5 repetitions of Note 3 and Note 4 within each call were analyzed for defining and comparing note types. Based on statistical comparisons of acoustic parameters among the four note types, the final note classification was determined.

Using the finalized note type classification, we further identified primary and higher-order acoustic units including note group and note series, respectively. The final acoustic analysis included all notes from all calls across all 13 recordings, with no restriction on the number of note repetitions. All 13 recordings were examined to assess the distribution of call variants across individuals and determine whether the observed structural diversity represented intra-individual or inter-individual variation.

### 2.3. Statistical Analysis

All analyses were performed in R v4.3.1, with statistical significance set at *p* < 0.05. Descriptive statistics are presented as mean ± standard deviation (SD). All acoustic data were tested for normality using the Shapiro–Wilk test and for homogeneity of variance using Levene’s test to determine whether parametric or non-parametric tests were appropriate.

To assess acoustic consistency within repetitive note types (Note 3 and Note 4), repeated-measures ANOVA was conducted on the first 5 consecutive repetitions of each type. Results revealed no significant differences among consecutive notes within each type (all *p* > 0.05), confirming acoustic consistency. Therefore, mean values of ND and DF were calculated to represent each Note 3 and Note 4 in subsequent analyses. Note-level acoustic parameters were then compared across the four initially identified note types (Note 1–4) using pairwise *t*-tests with Bonferroni correction. If note types showed no significant differences in both temporal and spectral parameters, they were merged into a single category. Primary and higher-order acoustic units (note groups and note series) were subsequently defined based on the composition and arrangement of the final note types. Within *Z. lishuiensis*, calls were classified based on note group variation observed in oscillograms. Temporal parameters among call variants were compared using Kruskal–Wallis tests, followed by Dunn’s post hoc test when significant.

Acoustic parameters of advertisement calls were compared between *Z. lishuiensis* and the previously reported *Z. zhoukaiyae* [24] using independent samples *t*-tests. The acoustic data of *Z. zhoukaiyae* were obtained from Fang et al. [24], where recordings were conducted in Dabie Mountain, Anhui Province (19–22 April 2016; air temperature: 10.7–13.7 °C; sampling rate: 44.1 kHz/16-bit). Both studies used the same sampling rate and analyzed calls with Praat software, ensuring methodological consistency. Additionally, we compiled acoustic parameters (CD and DF) from ten other *Zhangixalus* species with published acoustic data [20,21,22,23,25]. Recording site, air temperature, and SVL were also extracted when available for reference. These data were synthesized to characterize acoustic diversity within the genus *Zhangixalus*.

## 3. Results

**Figure 2:**
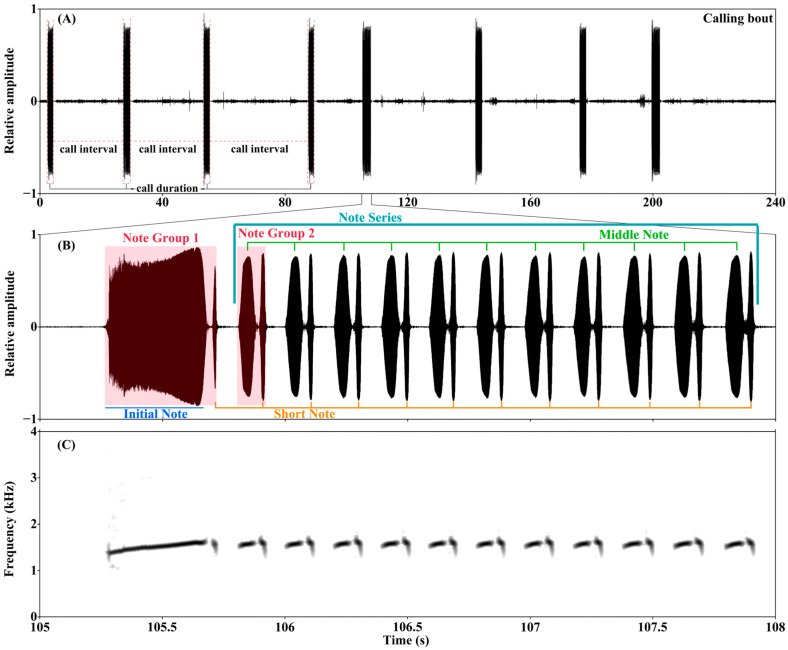
Acoustic characteristics of *Zhangixalus lishuiensis* advertisement calls. (**A**) Oscillogram of a 240 s calling bout (call voucher: ZL200406_001) recorded at 7.8 °C air temperature and 50.7 dB ambient noise level. (**B**) Oscillogram and (**C**) spectrogram of a single call showing the note structure: Note Group 1 (NG1) composed of an Initial Note and Short Notes; Note Group 2 (NG2) composed of Middle Notes and Short Notes, with consecutive NG2s forming a Note Series.

The advertisement calls of male *Z. lishuiensis* exhibit a hierarchical organization comprising distinct acoustic elements (Figure 2). During a typical calling bout, males produce a series of advertisement calls separated by call intervals (Figure 2A). Each individual call exhibits a consistent structural pattern composed of multiple notes organized into distinct groups (Figure 2B).

Through comparative analysis of ND and DF among the four initially identified note types, only Note 2 and Note 4 showed no significant differences in both ND and DF (both adjusted *p* > 0.05; Table 1), indicating they represent the same acoustic unit (Table 1). All other initial note types could be distinguished by at least one acoustic parameter (Table 1). Consequently, three distinct note types were finally identified: Initial Note (Note 1), Middle Note (Note 3), and Short Note (Note 2/4), distinguished. These notes are organized into two primary acoustic units: the first note group (Note Group 1, NG1) consists of an Initial Note and Short Note, and the second note group (Note Group 2, NG2) comprises a Middle Note and Short Note. Multiple NG2s are temporally arranged into a continuous Note Series (NS) (Figure 2B,C). The complete advertisement call consists of an NG1 followed by an NS, demonstrating a consistent structural pattern in this population (Figure 2B).

**Figure 3:**
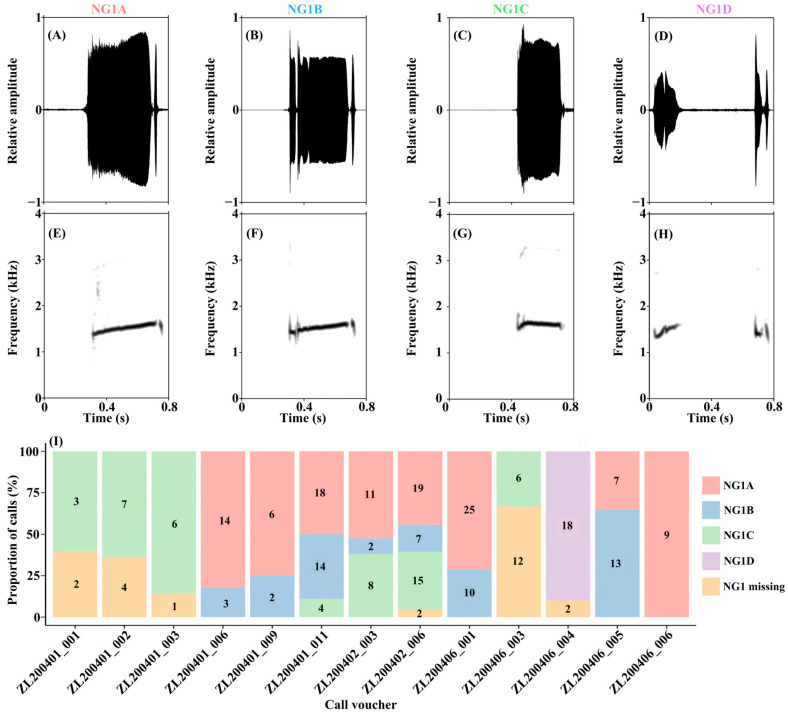
Variation in Note Group 1 (NG1) structure of *Zhangixalus lishuiensis* advertisement calls. (**A**–**D**) Oscillograms of four NG1 variants (NG1A, NG1B, NG1C, and NG1D, respectively); (**E**–**H**) corresponding spectrograms. (**I**) Proportion of NG1 variants and NG1 missing across individual call vouchers.

Among 227 calls of *Z. lishuiensis*, we identified four distinct NG1 structural variants (NG1A–D), with an additional 23 calls lacking NG1 entirely. The most common pattern (NG1A, *n* = 109) consists of a complete Initial Note followed by a Short Note (Figure 3A,E). NG1B (*n* = 51) features one or two shorter Initial Notes preceding this standard pattern (Figure 3B,F). NG1C (*n* = 49) represents a simplified form containing only the Initial Note without the Short Note (Figure 3C,G). NG1D (*n* = 18), apparently derived from NG1A, comprises only the onset and offset portions of the Initial Note (omitting the middle section) followed by a Short Note, creating a distinctive fragmented pattern (Figure 3D,H). Among the 13 recorded individuals in the LS population, only one produced calls containing exclusively NG1A, while the remaining 12 individuals emitted calls with various NG1 types. Notably, one individual produced calls representing all four call types (Figure 3I).

Furthermore, acoustic parameters, including CD, duration of NG1 (DNG1), interval between NG1 and NG2 (ING12), number of NG2 (NNG2), mean duration of NG2 (DNG2), mean interval between two NG2s (ING22), and duration of NS (DNS), differed significantly amongthe five call types (all *p* < 0.001) (Table 2). These findings demonstrate substantial intra-population variation in call structure, with NG1 composition serving as a primary source of acoustic diversity in the LS population.

The advertisement call structure of *Z. lishuiensis* shared fundamental similarities with that reported for the *Z. zhoukaiyae* [24], with both species producing calls consisting of a long-duration initial acoustic unit (analogous to NG1 in the *Z. lishuiensis*) followed by a series of shorter-duration units (analogous to NG2). However, notable differences exist in temporal organization: Z. zhoukaiyae notes are more continuous, whereas *Z. lishuiensis* produces more discrete, discontinuous notes. The call interval of *Z. lishuiensis* was 22.8 ± 16.1 s (Table 3). Despite these structural similarities, comparisons between species revealed significant acoustic differences (Table 3). *Z. lishuiensis* had significantly longer temporal parameters, including DNG1, ING12, DNG2, and ING22, than *Z. zhoukaiyae* (all *p* < 0.001). In contrast, *Z. zhoukaiyae* produced more NG2 repetitions (NNG2) and longer CD (both *p* < 0.05). The DF was significantly higher in *Z. zhoukaiyae* (*p* < 0.05).

Acoustic parameters compiled from eleven *Zhangixalus* species revealed substantial variation in call characteristics (Table 4). CD ranged over an order of magnitude, from 0.075 s in *Z. dugritei* to 2.31 s in *Z. zhoukaiyae*. DF exhibited a threefold range, from 0.828 kHz in *Z. omeimontis* to 2.679 kHz in *Z. achantharrhena*. Recording conditions also varied considerably, with temperatures ranging from 4.0 °C to 20.2 °C and SVL from 34.2 mm to 87.6 mm where reported.

## 4. Discussion

### 4.1. Acoustic Diversity in Z. lishuiensis and Its Potential Functions

In *Z. lishuiensis*, male advertisement calls exhibit a hierarchical structure composed of three note types (Initial Note, Middle Note, and Short Note), organized into two acoustic units (NG1 and NG2). Multiple NG2s are sequentially arranged to form a Note Series, representing the stereotyped call pattern. Compared to most reported *Zhangixalus* species [20,21,22,23,24,25], this call structure is relatively complex, showing similarity only to *Z. zhoukaiyae* [24]. Notably, considerable structural variation occurs within NG1, producing four distinct structural variants (NG1A–D). Importantly, this call diversity exists at the individual level; for example, individual ZL202402_006 exhibited four call variants, demonstrating intra-individual acoustic variation.

This structural diversification within NG1 may serve important communicative functions in complex acoustic environments, such as multi-species breeding choruses [37]. Previous studies demonstrated that the first long-duration note (analogous to NG1) of advertisement calls in *Z. zhoukaiyae* conveys sufficient information for individual recognition and plays a primary role in female mate choice [24,38]. Given this functional significance, the presence of multiple NG1 variants in *Z. lishuiensis* may represent adaptive modifications in male signal production that enhance signal recognition, maintain individual distinctiveness, or increase attractiveness under acoustically competitive conditions.

Among these variants, NG1D, which omits the central portion of the initial note, may represent an adaptive modification that enhances communication efficiency. One hypothesis is that this variant may exploit perceptual restoration mechanisms in receivers, whereby the auditory system reconstructs missing acoustic information masked by noise [39,40]. If female *Z. lishuiensis* possess similar perceptual capabilities, NG1D could potentially enhance call detectability under chorus conditions. However, evidence for such mechanisms remains inconsistent across anurans. Studies on *Dryophytes chrysoscelis* and *D. versicolor* found no support for auditory restoration enhancing call attractiveness [39,41], suggesting this mechanism may be species-specific or context-dependent. Alternatively, NG1D may simply represent a production error or energetic constraint rather than an adaptive signal design. Therefore, controlled playback experiments comparing female phonotactic responses to NG1D versus complete NG1 variants under varying noise conditions would be necessary to distinguish between these hypotheses and determine the functional significance, if any, of this structural variant in *Z. lishuiensis*.

### 4.2. Acoustic Divergence Between Z. lishuiensis and Z. zhoukaiyae

Although previous studies suggested that *Z. lishuiensis* and *Z. zhoukaiyae* exhibit minimal genetic and morphological differences [31,32,33], our acoustic comparison reveals significant divergence in key temporal and spectral acoustic parameters, despite overall structural similarity in advertisement calls between *Z. lishuiensis* and *Z. zhoukaiyae* [24]. *Z. lishuiensis* exhibited significantly longer temporal parameters, including DNG1, ING12, DNG2, and ING22; whereas *Z. zhoukaiyae* produced more NG2 repetitions and longer CD. Additionally, *Z. lishuiensis* displayed a lower DF.

The difference in DF is not only a key indicator for species recognition but may also partially reflect body size variation, as DF typically decreases with increasing body size in anurans, a phenomenon observed both at intra-specific and inter-specific levels [15,42,43]. Body size differences could potentially contribute to the DF variation between the two taxa. However, current morphometric data (*Z. lishuiensis*: *n* = 3, SVL = 34.2–36.2 mm; *Z. zhoukaiyae*: *n* = 6, SVL = 27.9–37.1 mm [29]) are limited in sample size and lack appropriate statistical comparison to establish this relationship conclusively. Additionally, environmental factors (e.g., temperature) may contribute to acoustic differences between closely related species. Temperature can differentially impact acoustic signal production in anurans, with many species showing temperature-dependent variation in temporal call parameters [12,44,45,46]. The recording temperature for *Z. lishuiensis* (7.0–7.8 °C) was lower than that for *Z. zhoukaiyae* (10.7–13.7 °C). However, *Z. lishuiensis* exhibited longer durations for individual acoustic units (e.g., DNG1, ING12, DNG2, and ING22), while *Z. zhoukaiyae* produced more NG2 repetitions, resulting in longer overall CD. This complex pattern suggests that temperature alone cannot explain the observed acoustic differences, which likely reflect species-level variation in call structure and organization.

Despite these clear acoustic distinctions, if the two species are indeed conspecific, the observed differences could represent population-level geographic variation in call parameters. Such geographic variation has been documented in other anurans such as two *Eleutherodactylus* frogs in Puerto Rico, where it is attributed to geographic isolation [47]. Similarly, in the European pool frog (*Rana lessonae*), regionally distinct acoustic patterns serve as phylogeographic indicators of population history and limited gene flow [48]. The low genetic divergence (0.9–1.6% mitochondrial DNA) [31,32,33] and minimal morphological differentiation [32] between *Z. lishuiensis* and *Z. zhoukaiyae* resemble patterns observed in cryptic species complexes where bioacoustic data proved critical for species delimitation (e.g., *Limnonectes* spp.) [35]. However, the present study is limited to two populations and lacks data on call consistency across geographic ranges or behavioral evidence of reproductive isolation. The observed acoustic divergence may represent either species-level diagnostic characters or population-level geographic variation. Although we lack decisive acoustic evidence to definitively resolve the taxonomic relationship between *Z. lishuiensis* and *Z. zhoukaiyae*, the acoustic divergence observed may carry phylogeographic significance, potentially reflecting historical isolation and local adaptation to different breeding environments that have shaped the evolutionary trajectories of these two taxa.

### 4.3. Call Diversity in the Genus Zhangixalus

The genus *Zhangixalus* exhibits considerable acoustic diversity. Our comparative results of eleven *Zhangixalus* species revealed substantial variation in CD and DF. This acoustic variation may reflect the influence of multiple factors. For example, body size differences may influence DF variation, as larger anurans typically produce lower-frequency calls [15,42,43]. Environmental conditions, including recording temperature, background noise and habitat characteristics, may also promote acoustic divergence [49,50,51]. Additionally, phylogenetic relationships may constrain or shape acoustic parameters, as demonstrated in other anuran groups where temporal call parameters exhibits strong phylogenetic signal (e.g., genus *Microhyla* [15]). Within this diversity, *Z. lishuiensis* and *Z. zhoukaiyae* share similar CD and DF, potentially reflecting their close phylogenetic relationship [31,32,33] or convergent adaptation to similar acoustic environments.

### 4.4. Future Directions

The diversification of call structure in *Z. lishuiensis* likely reflects a complex interplay of genetic divergence, phenotypic plasticity, and local ecological adaptation. However, several key questions remain to be addressed. First, do NG1 variants serve distinct communicative functions? Behavioral playback experiments are needed to determine whether the four variants (NG1A–D) differentially affect female preference or male-male competition, particularly under varying noise conditions. Second, what is the taxonomic relationship between *Z. lishuiensis* and *Z. zhoukaiyae*? Integrating multi-population acoustic sampling, common-garden and playback experiments, and nuclear genetic markers would provide the evidence necessary to determine their taxonomic status. Third, what mechanisms underlie call diversity across the genus *Zhangixalus*? Comprehensive bioacoustic surveys across the genus, integrating body size measurements, environmental variables, and phylogenetic relationships, would elucidate the internal and external factors driving acoustic diversification in this lineage.

## 5. Conclusions

This study presents the first detailed acoustic description of *Z. lishuiensis*, revealing four NG1 structural variants that demonstrate intra-individual acoustic diversity within the Lishui population. Despite overall structural similarity in advertisement calls, *Z. lishuiensis* differs significantly from *Z. zhoukaiyae* in temporal and spectral parameters, indicating acoustic divergence between these taxa. Comparative analysis across eleven *Zhangixalus* species revealed substantial variation in CD and DF, highlighting considerable acoustic diversity within this genus. The occurrence of multiple NG1 variants within individuals indicates acoustic flexibility that may enhance communication efficacy under variable conditions. These findings enrich the bioacoustic characterization of *Zhangixalus* and establish a foundation for acoustic-based species identification, monitoring, and taxonomic studies in this genus.

## Figures and Tables

**Table 1:** Pairwise comparisons of note duration (ND) and dominant frequency (DF) among the four initially identified note types using pairwise *t*-tests with Bonferroni correction. The upper triangle shows *t*-values and adjusted *p*-values for ND comparisons; the lower triangle shows *t*-values and *p*-values for DF comparisons. Bold values indicate statistical significance (α = 0.05).

	Note 1	Note 2	Note 3	Note 4
Note 1	ND: 0.323 ± 0.061 s DF: 1.53 ± 0.11 kHz	**t = 13.87,** **adjusted *p* < 0.001**	**t = 14.83,** **adjusted *p* < 0.001**	**t = 14.44,** **adjusted *p* < 0.001**
Note 2	t = −2.97, adjusted *p* = 0.124	ND: 0.022 ± 0.003 s DF: 1.58 ± 0.12 kHz	**t = −6.35,** **adjusted *p* < 0.01**	t = −2.12, adjusted *p* = 0.430
Note 3	**t = −4.30,** **adjusted *p* = 0.022**	t = 0.13, adjusted *p* = 1.000	ND: 0.047 ± 0.010 s DF: 1.58 ± 0.10 kHz	**t = 7.47,** **adjusted *p* < 0.001**
Note 4	**t = −6.59,** **adjusted *p* < 0.01**	t = −3.10, adjusted *p* = 0.104	**t = −5.16,** **adjusted *p* < 0.01**	ND: 0.025 ± 0.004 s DF: 1.60 ± 0.11 kHz

**Table 2:** Descriptive statistics, expressed as means ± SD and range, for call duration (CD), duration of Note Group 1 (DNG1), interval between Note Group 1 and 2 (ING12), number of Note Group 2 (NNG2), mean duration of Note Group 2 (DNG2), mean interval between two Note Group 2 (ING22), and duration of Note Series (DNS) in *Zhangixalus lishuiensis* based on the structure of Note Group 1. Results of Kruskal–Wallis tests with call types based on NG1 variation as the factor are given in the table. Means with different superscripts differ significantly (Dunn’s post hoc test, α = 0.05, a > b > c > d). NA: not applicable.

Type	N	CD (s)	DNG1 (s)	ING12 (s)	NNG2	DNG2 (s)	ING22 (s)	DNS (s)
NG1A	109	2.258 ± 0.794 ^a^ 0.815–3.632	0.373 ± 0.069 ^c^ 0.185–0.529	0.077 ± 0.022 ^c^ 0.017–0.124	10.9 ± 3.3 ^a^ 4–17	0.092 ± 0.015 ^ab^ 0.050–0.113	0.079 ± 0.021 ^b^ 0.032–0.115	1.983 ± 0.758 ^a^ 0.678–3.303
NG1B	51	2.177 ± 0.580 ^a^ 1.309–3.530	0.436 ± 0.083 ^b^ 0.213–0.553	0.080 ± 0.020 ^c^ 0.027–0.104	9.8 ± 2.9 ^ab^ 5–17	0.096 ± 0.016 ^a^ 0.056–0.111	0.083 ± 0.017 ^b^ 0.041–0.112	1.824 ± 0.530 ^a^ 1.075–3.041
NG1C	49	1.500 ± 0.532 ^b^ 0.632–3.197	0.307 ± 0.079 ^d^ 0.178–0.544	0.108 ± 0.056 ^a^ 0.011–0.179	7.7 ± 2.9 ^c^ 3–14	0.087 ± 0.019 ^bc^ 0.0366–0.125	0.067 ± 0.026 ^c^ 0.021–0.113	1.242 ± 0.484 ^b^ 0.402–2.871
NG1D	18	2.325 ± 0.409 ^a^ 1.567–3.021	0.754 ± 0.040 ^a^ 0.711–0.879	0.102 ± 0.012 ^b^ 0.092–0.140	8.8 ± 2.4 ^bc^ 4–13	0.087 ± 0.008 ^bc^ 0.061–0.094	0.089 ± 0.006 ^ab^ 0.082–0.110	1.635 ± 0.430 ^a^ 0.742–2.362
NG1 missing	23	1.626 ± 0.651 ^b^ 0433–3.229	NA	NA	9.4 ± 5.9 ^b^ 2–29	0.083 ± 0.012 ^c^ 0.051–0.095	0.093 ± 0.017 ^a^ 0.042–0.125	1.626 ± 0.652 ^a^ 0.433–3.229
Statistical results		χ^2^ = 49.4, *p* < 0.001	χ^2^ = 94.7, *p* < 0.001	χ^2^ = 32.0, *p* < 0.001	χ^2^ = 30.2, *p* < 0.001	χ^2^ = 19.2, *p* < 0.001	χ^2^ = 23.3, *p* < 0.001	χ^2^ = 41.0, *p* < 0.001

**Table 3:** Comparison of acoustic parameters in advertisement calls between *Zhangixalus lishuiensis* and *Zhangixalus zhoukaiyae*. Statistical comparisons were performed using Student’s *t*-tests. Data are presented as means ± SD. CI: call interval, CD: call duration, DF: dominant frequency, DNG1: duration of Note Group 1, ING12: interval between Note Group 1 and 2, NNG2: number of Note Group 2, DNG2: mean duration of Note Group 2, ING22: mean interval between two Note Group 2. NA: not applicable.

Acoustic Parameters	*Z. lishuiensis* (*n* = 13) (This Study)	*Z. zhoukaiyae* (*n* = 41) (Fang et al. [24])	Statistical Result
CI (s)	22.8 ± 16.1	NA	
CD (s)	1.96 ± 0.49	2.31 ± 0.65	t = −2.06, *p* = 0.044
DF (kHz)	1.515 ± 0.083	1.574 ± 0.023	t = −2.53, *p* = 0.014
DNG1 (s)	0.383 ± 0.129	0.196 ± 0.039	t = 5.15, *p* < 0.001
ING12 (s)	0.105 ± 0.040	0.048 ± 0.017	t = 5.00, *p* < 0.001
NNG2	9.1 ± 2.1	19.0 ± 4.7	t = −10.57, *p* < 0.001
DNG2 (s)	0.093 ± 0.013	0.064 ± 0.002	t = 8.01, *p* < 0.001
ING22 (s)	0.084 ± 0.017	0.047 ± 0.001	t = 7.84, *p* < 0.001

**Table 4:** Acoustic parameters of advertisement calls in *Zhangixalus* species from published studies. Call duration (CD), dominant frequency (DF), air temperature (AT), and snout-vent length (SVL) are presented as mean ± SD or range. NA: not applicable.

Species [Reference]	Recording Site	CD (s)	DF (kHz)	AT (°C)	SVL (mm)
*Z. achantharrhena* [25]	Mt. Singgalang, Balingka, West Sumatra, Indonesia	0.3 ± 0.01	1.638–2.679	NA	NA
*Z. chenfui* [20]	Daiguocao, Mount Wawu, Sichuan Province, China	0.418 ± 0.055	2.00 ± 0.08	17.6	NA
*Z. chenfui* [22]	Mt. Emei-shan, Sichuan Province, China	0.158–0.645	2.100–2.3488	13.0	NA
*Z. dennysi* [21]	Mt. Diaoluo National Nature Reserve, Hainan Province, China	0.2355	1.268 ± 0.076	NA	87.6 ± 5.3
*Z. dugritei* [20]	Daiguocao, Mount Wawu, Sichuan Province, China	1.238 ± 0.046	1.70 ± 0.05	13–16	NA
*Z. dugritei* [22]	Mt. Wa-shan, Sichuan Province, China	0.075–1.015	1.45–2.5	4.0–8.0	NA
*Z. dulitensis* [25]	Ulu Temburong National Park, Brunei	0.11 ± 0.01	1.549–3.336	NA	NA
*Z. faritsalhadii* [25]	Mt. Slamet, Kalipagu, Ketenger Village, Central Java Province, Indonesia	0.1 ± 0.04	1.338–1.581	19.5–20.2	37.6
*Z. lishuiensis* (this study)	Liandu Fengyuan Provincial Nature Reserve, Lishui, Zhejiang Province, China	1.96 ± 0.49	1.515 ± 0.083	7.0–7.8	34.2–36.2
*Z. omeimontis* [22]	Mt. Emei-shan, Sichuan Province, China	0.166–0.460	0.828–0.977	11.0–13.0	NA
*Z. pinglongensis* [23]	Shiwandashan National Nature Reserve, Guangxi Province, China	0.43–0.47	1.6–3.0	18.4	38.2
*Z. prominanus* [25]	Telekom Loop, Fraser’s Hill, Pahang, Malaysia	0.1–0.4	NA	NA	NA
*Z. zhoukaiyae* [24]	Dabie Mountain, Anhui Province, China	2.31 ± 0.65	1.574 ± 0.023	10.7–13.7	NA

## Data Availability

The raw data and R codes in this article are available from the figshare https://doi.org/10.6084/m9.figshare.28418261. Figure 1A was created by the authors using elevation data from the WorldClim version 2 database (http://www.worldclim.org/) and administrative boundary data based on the Chinese standard map (review number GS(2024)0650), which was obtained from the official website of the National Platform for Common GeoSpatial Information Services (https://www.tianditu.gov.cn). All data are freely available for research purposes with no copyright concerns.

## Abbreviations

The following abbreviations are used in this manuscript:

CD	Call duration
CI	Call interval
DF	Dominant frequency
DNG1	Duration of Note Group 1
DNG2	Mean duration of Note Group 2
DNS	Duration of Note Series
FFT	Fast Fourier transform
ING12	Interval between Note Group 1 and 2
ING22	Mean interval between two Note Group 2
NG1	Note Group 1 (first note group)
NG2	Note Group 2 (second note group)
NNG2	Number of Note Group 2
NS	Note Series
NA	Not applicable
SD	Standard deviation
SVL	Snout–vent length
